# Beyond Visual Observations: Establishing the Mechanical Stability Threshold of Nanothin Polyethylene Layers

**DOI:** 10.3390/membranes16020072

**Published:** 2026-02-20

**Authors:** Alfonso Lemus-Solorio, Mariana Ramos-Estrada, Salomón R. Vásquez-García, José L. Rivera

**Affiliations:** 1 Department of Chemical Engineering, Universidad Michoacana de San Nicolás de Hidalgo, Morelia 58000, Mexico; 1209689x@umich.mx (A.L.-S.); mariana.ramos@umich.mx (M.R.-E.); salomon.vasquez@umich.mx (S.R.V.-G.); 2 Department of Physico-Mathematical Sciences, Universidad Michoacana de San Nicolás de Hidalgo, Morelia 58000, Mexico

**Keywords:** polyethylene, film, ultrathin, disjoining pressure, molecular dynamics

## Abstract

This paper investigates the mechanical stability and critical thickness of free-standing, ultrathin molten polyethylene films using Molecular Dynamics simulations. By comparing the “interfacial drying” and “film stretching” methodologies, this research establishes that both methods consistently identify a stability threshold where continuous films transition into fibrillar and void structures known as “crazes”. A key finding is that films at extremely reduced thicknesses exhibit an anisotropic pressure profile in their core—characterized by a positive normal pressure—which serves as a manifestation of positive disjoining pressure and a precursor to film transformation. Consequently, the study proposes a more rigorous stability criterion based on mechanical isotropy, which yields higher critical thickness values (approximately 6.5 nm at 373.15 K and 9.3 nm at 673.15 K) than those previously estimated from short-term (100 ns) visual observations. Ultimately, the work concludes that maintaining a negative disjoining pressure is fundamental to the structural integrity of these polymeric nanomaterials.

## 1. Introduction

In the contemporary landscape of materials science, the drive toward miniaturization has pushed polymeric materials into regimes where their physical dimensions approach the molecular scale. Ultrathin polymer films, typically defined as having thicknesses below 100 nm, are no longer merely passive components; they are essential functional elements in technologies ranging from organic electronics [1,2,3] and gas-separation membranes [4,5,6] to advanced coatings for aerospace applications [7,8,9]. However, as the thickness of a polymer film decreases, its behavior begins to deviate drastically from its bulk counterparts. This departure is driven by the increasing dominance of interfacial effects, where the ratio of surface-to-volume atoms becomes so high that the properties of the entire material are dictated by the boundaries rather than the interior.

Polyethylene, the most widely used plastic globally, serves as a fundamental model for understanding these phenomena due to its chemically simple structure. Despite its simplicity, the physicochemical behavior of polyethylene in the form of free-standing, ultrathin films remains a subject of intense scientific inquiry [10,11,12,13,14]. At thicknesses of only a few nanometers, these films exhibit unique phase transitions [15,16], altered glass transition temperatures [17], and, most critically, a significant susceptibility to mechanical instability [18]. Understanding the fundamental limits of how thin a polyethylene film can become before it spontaneously ruptures is not only a theoretical challenge but also a practical requirement for the design of stable nanomaterials [19].

The stability of any thin liquid or molten film is governed by a competition between stabilizing and destabilizing forces. In macroscopic systems, the primary restoring force is surface tension, which acts to minimize the surface area of the film. However, in the nanoscopic regime, a second, more complex phenomenon emerges: disjoining pressure [20]. It represents the additional pressure that arises when two interfaces are brought close enough together that their interaction zones overlap. For a free-standing film in a vacuum, these two interfaces are the top and bottom surfaces of the polymer layer. When the film is thick, the two interfaces “see” only the bulk polymer between them, and the disjoining pressure is zero. As the film thins to the nanometer scale, the attractive van der Waals forces between the two interfaces can create a “negative” disjoining pressure that helps maintain film integrity, whereas at ultra-reduced thickness scales, the emergence of “positive” disjoining pressure induces interfacial separation, leading to spontaneous rupture or “dewetting” in supported films.

While disjoining pressure has been extensively studied in the context of thin liquid films supported on solid substrates [21,22,23], its role in free-standing molten polymer films is less understood [24]. In these systems, the absence of a supporting substrate means the film must rely entirely on its internal cohesive energy and the balance of local pressures to maintain a continuous structure.

Experimentally determining the “critical thickness”, the absolute minimum thickness at which a film remains stable, is subject to substantial technical constraints. Stretching a polymer film to its breaking point in a lab occurs over timescales of seconds [11], which are vastly different from the molecular timescales of chain relaxation. Furthermore, macroscopic environmental factors, such as capillary waves and mechanical vibrations, often trigger rupture before the fundamental physical limit of the material is reached [25].

This is where Molecular Dynamics (MD) simulations become an indispensable tool. By simulating the system at the atomic or united-atom level, researchers can isolate the fundamental intermolecular forces that govern stability [14,18]. Previous computational efforts have utilized methods like “interfacial drying” (removing molecules one by one) [14] or “film stretching” (expanding the simulation box) to probe these limits [26]. However, these studies have often yielded conflicting results regarding the exact value of the critical thickness. For instance, recent mesoscopic simulations suggested higher stability for certain polymers than united-atom models predicted for polyethylene [26]. This discrepancy highlights a critical need for a more rigorous mechanical criterion for stability—one that moves beyond simply observing whether a film ruptures during a short simulation window.

In an ultrathin film, the entire material effectively becomes an “interfacial region”. If the film is too thin, the normal pressure in the core of the film may fail to equilibrate with the vacuum, resulting in a positive disjoining pressure. By identifying the thickness at which the central core of the film regains mechanical isotropy—where the normal and lateral pressure components converge—a more precise and physically grounded “mechanical stability thickness” can be defined.

In this work, we carried out MD simulations to study the differences in the interfacial drying and stretching methodologies used to predict the “critical thicknesses” of polyethylene films by visual observations based on visual inspections over 100 ns trajectories, at temperatures in the range of 373.15–673.15 K. Mechanical analyses at the proposed “critical thicknesses,” obtained by visual observations and wider thicknesses, allowed us to propose a more physically grounded criteria for the limit thickness of stability.

## 2. Methodology

The self-standing film–vacuum interfaces of melted polyethylene chains were studied directly through MD simulations of polyethylene films surrounded by a vacuum. Systems of different thicknesses were simulated at both temperatures, 373.15 and 673.15 K. The simulation cells consisted of periodic parallelepipeds with two square sides and an interfacial area $A_{i}=l_{x}\times l_{y}=145\times 145{Å}^{2}$, which was large enough to contain stretched C_200_H_402_ chains in each lateral direction and contained the film’s surface. Due to their periodicity, the simulation cells had a variable length in the inhomogeneous (normal to the polymer surface) direction, large enough to contain the variable film thicknesses, with vacuum regions large enough to prevent artificial film–film interactions.

The initial systems consisted of ordered, stretched, and oriented chains that were parallel to the interfacial surface, with a chain–chain separation of 5 Å in all directions, while the ends of the chains were not paired with each other to ensure the mixing of the chains in the early stages of the simulation. If the chains do not mix, a film is not formed; instead, a fibril or a spherical aggregate develops. The initial positions of the sites in the configuration were obtained using an in-house code, which also randomly distributed atomic velocities. The chains were brought to the film–vacuum equilibrium slowly over a period of 1 ns, and the methods of interfacial drying [14,18] and film stretching [26] were applied in the NVT (constant number of molecules, rigid volume of simulation cell, and constant average temperature) canonical ensemble, with a timestep of 1 fs. The thermostat used was that of Nosé [27], implemented in the Large-scale Atomic/Molecular Massively Parallel Simulator (LAMMPS) [28].

**Figure 1:**
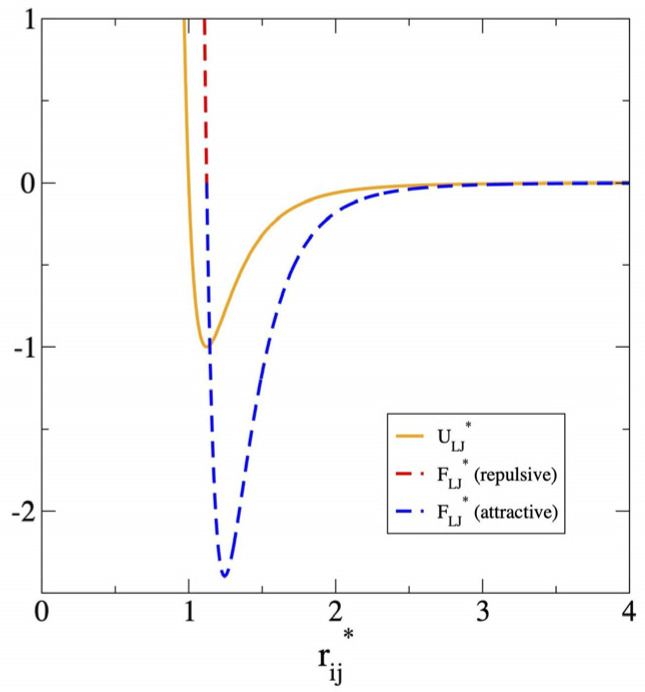
Reduced energy ($U_{LJ}^{*}=U_{LJ}/\epsilon_{ij}$) and reduced force ($F_{LJ}^{*}=F_{LJ}/\epsilon_{ij}$) of the Lennard–Jones potential as a function of the reduced separation ($r_{ij}^{*}=r_{ij}/\sigma_{ij}$) between interaction sites i and j.

The polyethylene chains interacted through the TraPPE potential [29], which is a united atom potential that considers the CH_3_ and CH_2_ functional groups of the polyethylene chains as Lennard–Jones sites of interaction. The non-bonded Lennard–Jones sites i and j, interacted through the following potential for energy ($U_{ij}$) and forces ($F_{ij}$)

(1) $$U_{LJ}=4\epsilon_{ij}\left[{\left(\frac{\sigma_{ij}}{r_{ij}}\right)}^{12}-{\left(\frac{\sigma_{ij}}{r_{ij}}\right)}^{6}\right]$$

(2) $$F_{LJ}=\frac{24\epsilon_{ij}}{r_{ij}}\left[2{\left(\frac{\sigma_{ij}}{r_{ij}}\right)}^{12}-{\left(\frac{\sigma_{ij}}{r_{ij}}\right)}^{6}\right]$$

where $r_{ij}$ represents the separation between Lennard–Jones sites i and j. $\sigma_{ij}$ and $\epsilon_{ij}$ are Lennard–Jones parameters, dependent on the interaction site; for unlike sites, standard arithmetic ($\sigma_{ij}$) and geometric ($\epsilon_{ij}$) combining rules were used. We used a long cutoff radius ($r_{c}=7.5\;\sigma_{ij}$) to consider all significant interactions and avoid the use of long-range corrections at the end of the simulations, or other approximations during the simulation, which mask the true dynamics of the system [30,31]. The Lennard–Jones potential is composed of long-range van der Waals attraction forces (dispersion) and short-range repulsive forces (Figure 1), which produce a valley in the energy profile, and at this minimum point in the energy, the force is zero; below this point, forces are repulsive, and beyond this point, they are attractive.

The TraPPE potential for linear alkanes uses harmonic potentials for interactions due to bond and angle bendings and a cosine function on the bending of dihedral angles [32]. The TraPPE potential has been employed to study the structural conformations of single polyethylene chains (C_1000_) [33], semicrystalline polyethylene (C_112_) [34], melted polyethylene chains (C_30_–C_150_) [35] and polyethylene blends (C_320_) [36]. The use of the TraPPE potential to study the crystallization process of polyethylene chains (C_192_) has produced results that are in better agreement with experiments compared to predictions of other interaction potentials [37].

Mechanical stability of the polyethylene films was studied through its pressure profiles, which were distributed locally in small slabs along the inhomogeneous direction. The profiles were calculated using Harasima contours [38], a feature available in LAMMPS [39], which arbitrarily distributes the stress from two interacting sites between the two originating slabs. While there is no unique definition for the local pressure, Harasima contours correctly describe interfacial pressures as the Irving–Kirkwood contours [40] in planar interfaces; however, they are problematic for curved interfaces [41,42].

## 3. Results

**Figure 2:**
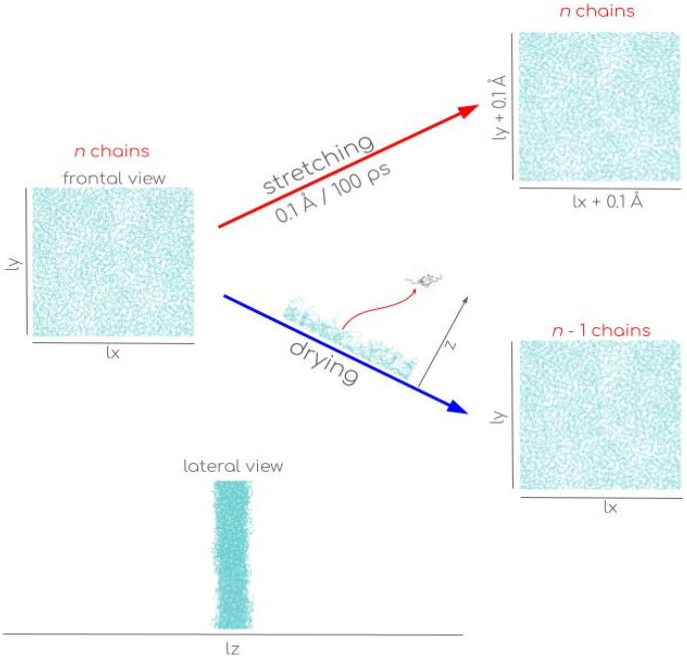
Schematic of the stretching and interfacial drying processes of a molten polyethylene chain film, with the aim of reducing the film thickness to $\tau_{lim}$. The cyan-colored lines represent C_200_H_402_ chains made of polyethylene.

Recent mesoscopic MD simulations on polyethylene glycol films have explored the minimum stability thickness, $\tau_{lim}$, using the stretching method (Figure 2) [26]. These studies have identified a $\tau_{lim}$ lower than the predictions obtained with united atom models for polyethylene films using the interfacial drying method (Figure 2) [14]. Despite the chemical differences between these polymers, one characterized by polar groups and the other by non-polar groups, it has been proposed that the controlled stretching method minimizes disturbances in the interfacial region, reducing undesirable fluctuations that compromise the integrity of the film [26]. To compare both approaches, polyethylene films were analyzed starting from configurations obtained by the interfacial drying method, successively removing interfacial chains until reaching the $\tau_{lim}$, which has been previously reported by our group for the range of 373.15 to 673.15 K. The final systems at the $\tau_{lim}$ correspond to ultrathin films with thickness in the range of (3.46–5.63 nm) at temperatures in the range of (373.15–673.15 K), which correspond to surface densities between 44 (373.15 K) and 55 (673.15 K) C_200_H_402_ chains per 100 nm^2^. Under these conditions, polyethylene remains in a molten state, which is due to the known shifts in melting temperatures [15,16] and vitreous transition temperatures [17,43] for nanoscopically thick films.

Using the configurations obtained by the interfacial drying method at $\tau_{lim}$ for both temperatures (373.15 and 673.15 K), the stretching method was applied to verify consistency with the results from the interfacial drying method. The process consisted of the gradual expansion of the simulation cell in the lateral directions, which contained the film surface, and this process also stretched the film surface. To avoid artificial instabilities, the stretching was performed at a rate of 0.1 Å per 100 ps (~0.07% ps^−1^), meaning that every 100 ps, the sides of the simulation cell were increased by 0.1 Å and the positions of the chain atoms within the simulation cell were adjusted proportionally. This stretching rate was limited only by current computing capabilities. Although this rate is much higher than experimental timescales, where stretching rates range from 0.7% to 2% in periods of seconds [11]. The small area of the periodically simulated system (145 × 145 Å^2^) allows the application of deformation forces without the interference of capillary waves, which are usually the main mechanism of rupture in macroscopic surfaces [25].

**Figure 3:**
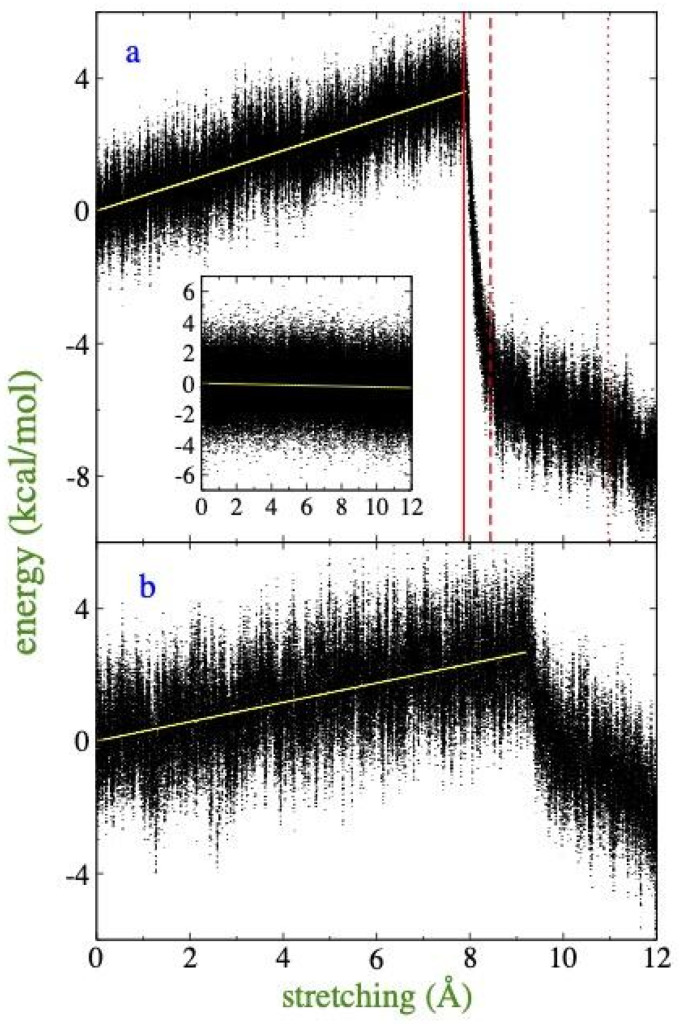
Change in the ${\Delta E}_{tot}$ of the polyethylene film as a function of degree of strain. Films are stretched departing from a configuration representing the $\tau_{lim}$ using the interfacial drying method, with a cell length of 14.5 nm in both lateral directions, stretched to a final length of 15.7 nm. The entire stretch (1.2 nm) was carried out in 12 ns. Each point on the profile was measured every 100 fs. The results correspond to temperatures of (**a**) 373.15 K and (**b**) 673.15 K. The yellow lines represent linear regressions, while the solid, dashed, and dotted red lines represent the stretches corresponding to the visualizations shown in Figure 4b,d,e, respectively. The inset figure represents the changes in vibrational energy at 373.15 K.

Despite being a dynamic process, the results of stretching are presented as a function of the change in the total energy of the system (${\Delta E}_{tot}$) with respect to the transverse stretched lengths of the simulation cell (*l_x,y_*), instead of time. This approach is valid since the low strain rate prevents significant artificial perturbations. The reference point to calculate ${\Delta E}_{tot}$ is the average energy of the film in the initial unstretched simulation cell (*l_x,y_*= 14.5 nm) obtained by the interfacial drying method at its $\tau_{lim}$. Figure 3a,b show the evolution of ${\Delta E}_{tot}$ at 373.15 and 673.15 K, respectively. For both temperatures, the maximum stretch of the film was approximately 8.28% carried out over a period of 12 ns. It is observed at both temperatures that the ${\Delta E}_{tot}$ increases linearly with the degree of deformation of the film, reflecting a progressive loss of stability and cohesion in the film as it moves away from its original unstretched equilibrium configuration. This linear correlation breaks down abruptly at stretches of approximately 0.8 nm at 373.15 K and 0.92 nm at 673.15 K, points at which a sudden drop is recorded in ${\Delta E}_{tot}$. This change corresponds to a spontaneous process of energy minimization and signals a structural transition: the rupture of the continuous film and its reorganization into more cohesive and thermodynamically stable conformations. This phenomenon is consistent with previous reports on polymeric systems describing the formation of “crazes” or fine cracks [44,45,46], where the material transforms into a network of nanometric fibrils separated by voids between 2 and 20 nm. Notably, the vibrational energy analysis (Figure 3a, inset) confirms that this sudden change is due exclusively to variations in interchain interaction energy, without alterations in the internal structure of the molecules. Furthermore, the higher energy required for rupture at 373.15 K, compared to 673.15 K, is attributed to the fact that the cohesive energy of systems in equilibrium naturally decreases with increasing temperature.

**Figure 4:**
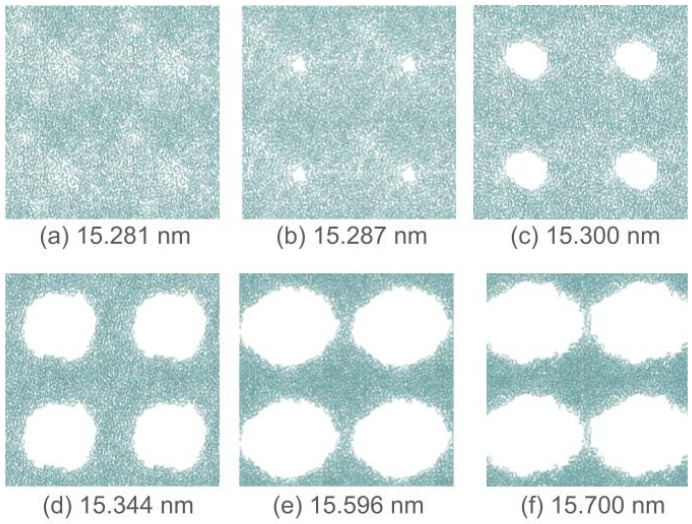
Frontal views of a polyethylene film simulated using classical MD simulations at 373.15 K (central cell with its first periodic surface images), where the length of the simulation cell has been stretched from 14.500 nm to the values reported in each figure. During the stretching process, the film becomes unstable, and surface cracking occurs on the polymer surface.

Frontal views of the polymer surface through the stretching process at 373.15 K are shown in Figure 4. The film evolves into a fibrillar structure that widens in the normal direction, while empty spaces expand laterally. The snapshots at key stages of the transformation demonstrate that the film transformation occurs when film pores develop and reach a critical size (Figure 4b) that prevents self-repair by diffusion of the chains. Small and transient pores (Figure 4a) develop and repair commonly in ultrathin films [14]. The transient appearance of these small pores can be useful as adsorption sites for light gases [47,48,49]. The unrepaired pores grow rapidly with further small stretches of the film (Figure 4c,d). The snapshots show that the drastic energy drop observed in the time profile of ${\Delta E}_{tot}$ (Figure 3a,b) ends only when the voids start to interconnect laterally (Figure 4e), stabilizing the system in a defined fibril configuration.

**Figure 5:**
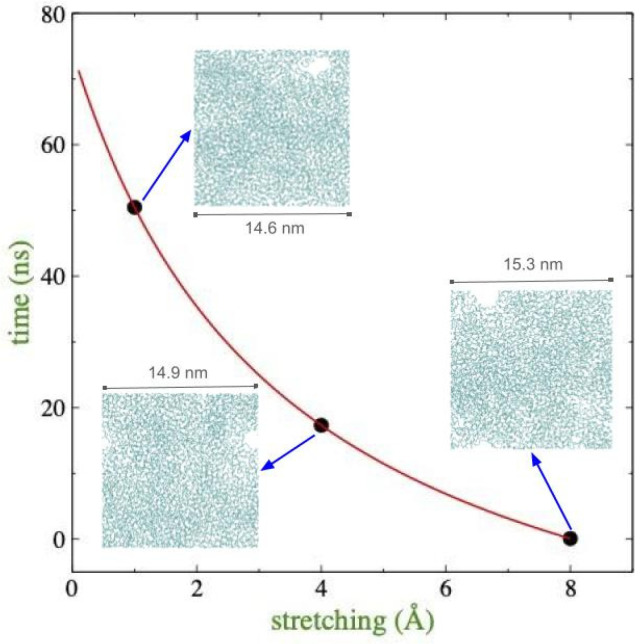
Time taken for polyethylene films, stretched beyond the $\tau_{lim}$ obtained using the interfacial drying method, to form pores that cannot be repaired and eventually led to films with fine surface cracks (crazes), composed of voids and fibrils. The plotted values represent average values from three simulations, started with different initial sets of velocities but the same atomic positions. The red line represents the fit to a function inversely dependent on the degree of deformation. The visualizations correspond to staged MD simulations where the first pore forms and fails to self-repair.

To validate the stability of the stretched configurations prior to the formation of “crazes”, the systems were allowed to evolve for 100 ns at the stretched lengths of the simulation cells, a standard criterion used in previous interfacial drying studies [14,31]. Simulation cells, stretched from 1 to 8 Å at 373.15 K, were evaluated in triplicate experiments, and in no case was the film integrity maintained during the test period. Regardless of the degree of stretching, the system invariably developed irreparable pores that transformed the film into fibrils (Figure 5). The time to the appearance of the first rupture pore showed an inverse decay, inversely proportional to the degree of the film deformation, which agrees with the observed changes in ${\Delta E}_{tot}$ in the linear regime (Figure 3), suggesting that as the film is stretched, progressively smaller perturbations in the stretched films will induce the transformation of the ultrathin film into more stable structures.

**Figure 6:**
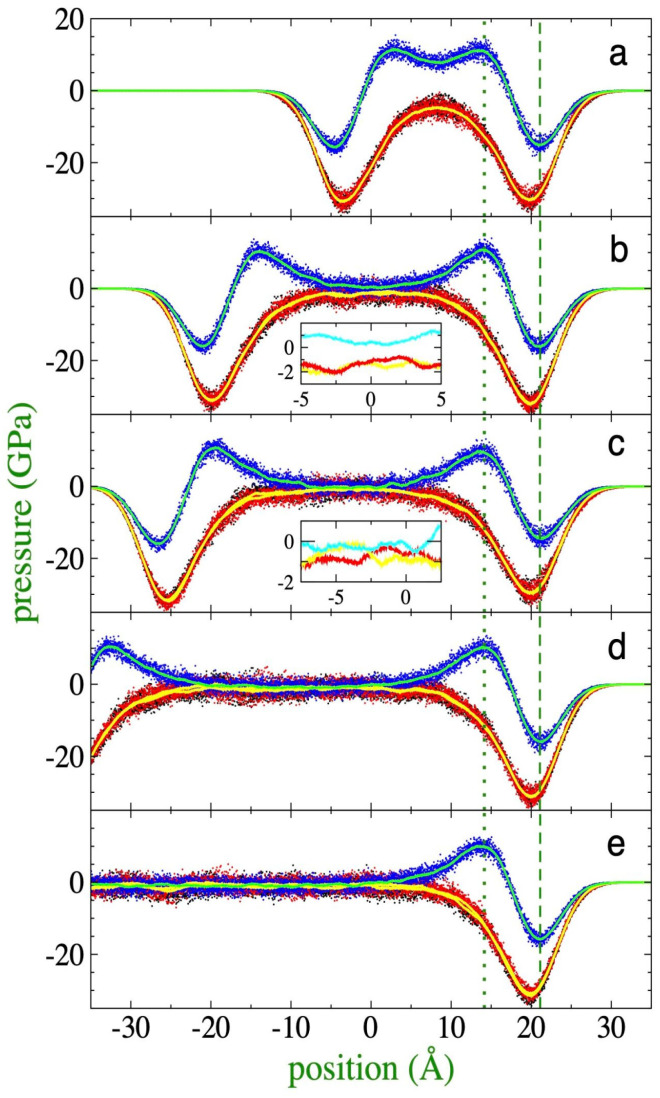
Pressure profiles of polyethylene films melted at 373.15 K, as a function of position within the simulation cell in the normal direction. The films contained (**a**) 94, (**b**) 155, (**c**) 176, (**d**) 224, and (**e**) 288 C_200_H_402_ polyethylene chains. Red, black, and blue dots represent the profiles in the *x* (lateral), *y* (lateral), and *z* (normal) directions, respectively. Solid yellow and cyan curves represent averages of the lateral and normal profiles, respectively. Green vertical lines represent the position of the maximum and minimum in the normal profiles. The inner graphs represent magnified views of the corresponding profile averages. All profiles were aligned to the 176-chain profile.

**Figure 7:**
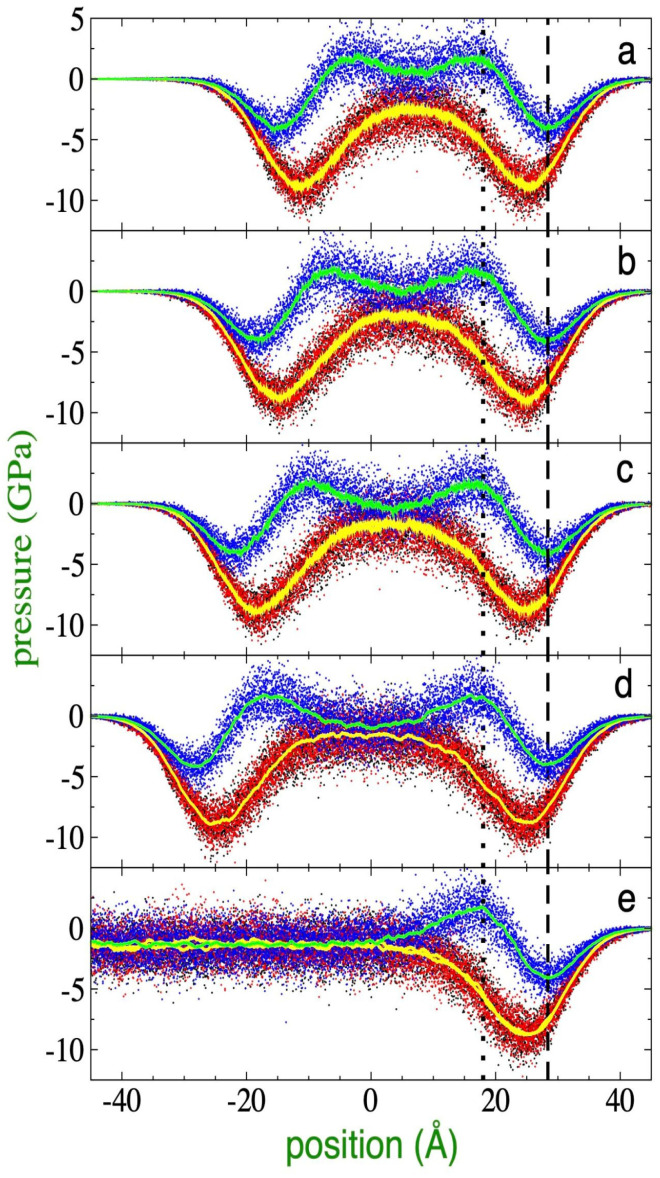
Pressure profiles of polyethylene films melted at 673.15 K, as a function of position within the simulation cell in the normal direction. The films contained (**a**) 116, (**b**) 125, (**c**) 135, (**d**) 155, and (**e**) 288 C_200_H_402_ polyethylene chains. Red, black, and blue dots represent the profiles in the *x* (lateral), *y* (lateral), and *z* (normal) directions, respectively. Solid yellow and cyan curves represent averages of the lateral and normal profiles, respectively. Black vertical lines represent the position of the maximum and minimum in the normal profiles. All profiles were aligned to the 155-chain profile.

MD simulations to define $\tau_{lim}$ of films using both the interfacial drying [14] and stretching [26] methods employ simulation periods on the ns scale as a standard criterion, carried out under the premise that no transition to fibril and void structures occurred during that interval. However, this observation time might prove insufficient to extrapolate the behavior to macroscopic surfaces. An additional critical factor is that films at thicknesses corresponding to $\tau_{lim}$ exhibit anisotropic pressure profiles in their central region (Figure 6a and Figure 7a), suggesting a possible latent mechanical instability on timescales longer than those that can be simulated.

With the aim of establishing a more rigorous criterion for mechanical stability, the thicknesses at which anisotropy disappears in the pressure profiles were determined. As observed in Figure 6a (373.15 K) and Figure 7a (673.15 K), the two lateral pressure profiles ($P_{lat}$) are equivalent to each other at both temperatures; however, normal pressure profiles ($P_{nor}$) at the center of the film do not converge with the lateral components. In fact, at this thickness, the values of $P_{nor}$ are positive, contrasting with the negative values of $P_{lat}$. Since regions with negative pressures, or negative forces (Figure 1), indicate cohesive and stable regions. Therefore, the pressure profiles showed lateral stability but revealed a latent instability in the normal direction. This behavior is characteristic of the phenomenon named disjoining pressure [20,50,51], in which interfacial regions of very thin films interact due to their proximity, inducing changes in the rest of the pressure profiles. Due to the disjoining pressures, the $P_{nor}$ of ultrathin films differs from the pressure of the surrounding medium (vacuum, P = 0), in contrast with the behavior of very thick films where pressures are in equilibrium. However, films as thin as 100 nm, dominated by van der Waals interactions, have been reported to maintain negative disjoining pressures [52,53]. Below a critical value, in the range of a few nm, the component $P_{nor}$ becomes positive, eventually probably inducing the transformation of the continuous film into fibril structures and void spaces at long timescales.

Conversely, when analyzing films with a higher number of chains (although still below the 100 nm threshold), such as the 288-chain systems illustrated in Figure 6e (373.15 K) and Figure 7e (673.15 K), it is observed that the pressures in the film core maintain isotropic behavior and negative values. This condition results in a negative disjoining pressure, which underlies the mechanical stability of these systems. While the 224-chain systems retain similar properties (Figure 6d and Figure 7d), starting with films with 176 chains (373.15 K), the beginning of the loss of isotropy becomes evident; at this point, the profile of $P_{nor}$ becomes less negative, and for the $P_{lat}$, the film seems to behave as formed by two monolayers, and those monolayers exhibit alternating pressure values similar to those of the $P_{nor}$ while the other remains more negative. The behavior of the $P_{nor}$ is critically accentuated in the 155-chain system, where $P_{nor}$ reaches positive values in the central region, while the $P_{lat}$ remain negative, a divergence that becomes even more pronounced when the thickness is reduced to 94 chains.

Considering the full extension of interfacial regions, the 176-chain system (surface density of ~84 chains/100 nm^2^) at 373.15 K represents the critical point where significant divergences emerge between the components $P_{nor}$ and $P_{lat}$ in the core of the film. Under these conditions, a thickness of ~6.5 nm is recorded, a value significantly higher than the 3.46 nm initially proposed as the “critical thickness” at which the film becomes unstable in previous studies using the 100 ns visual criterion of stability [14]. Obtaining films with such dimensions experimentally presents considerable technical challenges; stretching processes are typically performed using macroscopic surfaces, stretched on timescales of seconds and in multistages to mitigate structural disturbances, which results in macroscopic polyethylene films with a high aspect ratio near 10^8^ [11]. The minimum critical thicknesses reported in the experimental literature (~12 nm) [11] are almost double the new findings of this work. However, it is expected that experimental thicknesses can be further minimized through the development of new technology to achieve thinner dimensions [54,55,56]. The use of longer polymer chains can reduce the value of the predicted critical thickness, as multiple studies found an increment in the stability of polymeric systems when longer polymer chains are employed [57,58].

It was observed that the interfacial regions maintain consistent structural and mechanical characteristics (pressure profiles), regardless of the film thickness. When analyzing the points where the profiles of $P_{nor}$ reached their interfacial peaks and valleys, it was determined that both their location and magnitude remain practically unchanged across the different systems. This behavior is consistent with previous findings on surface tension in thin polyethylene and atomic self-standing films [14,31], which shows an independence from film thickness for the sub-100 nm simulated films.

At 673.15 K, an analogous behavior to that reported for 373.15 K is observed. Under these conditions, the system requires a minimum of 116 chains to preserve its structural integrity during the 100 ns criterion. However, at this “critical thickness”, the film exhibits a positive disjoining pressure, smaller than that corresponding to the profiles at 373.15 K, indicating also a marginal stability. As the number of chains increases, a progressive decrease in the profile values is recorded and $P_{nor}$ transitions from positive to negative values until reaching a condition of isotropy at greater thicknesses. An extrapolation analysis of the trend in the first four systems (from 116 to 155 chains) reveals that mechanical isotropy is formally established upon reaching a surface density of approximately 78 chains/100 nm^2^ (equivalent to a simulated system with 165 chains), which defines a mechanical stability thickness of 9.3 nm for this temperature.

## 4. Conclusions

The study demonstrates the consistency between interfacial drying and stretching methods for identifying the stability threshold thickness in ultrathin molten polyethylene films, confirming that film transformation occurs through the formation of pores that evolve into fibrillar and void structures. Through MD simulations, it was observed that stretching the simulation cell, and implicitly the polymer film itself, causes a linear increase in total energy until a critical point of instability is reached, where a sudden drop in energy signals the reorganization of the material into more thermodynamically stable conformations, as those conformations formed by fibrils and voids, named “crazes”.

Analysis of pressure profiles revealed that, at reduced thicknesses, in the limit of stability, observed using the 100 ns criterion, the film core exhibits marked anisotropy, with positive normal pressure and negative lateral pressures. This provides direct evidence of the positive disjoining pressure that precedes fragmentation of the continuous layer. At thicker film thicknesses, the development of mechanical isotropy can be established as a more rigorous stability criterion. Critical thicknesses of approximately 6.5 nm at 373.15 K and 9.3 nm at 673.15 K were determined under this new criterion, values that exceed previous estimates based solely on short-term structural integrity. Ultimately, this work concludes that the stability of these self-standing films in a vacuum depends on maintaining a negative disjoining pressure in the core, which imposes fundamental physical limits on the miniaturization of polymeric nanomaterials.

Future work aims to study the effects of the adsorption of light gas molecules on film stability in terms of the isotropy of the system’s pressure components.

## Data Availability

The data presented in this study are available on request from the corresponding author.
